# Immunoglobulin replacement therapy in antineutrophil cytoplasmic antibody-associated vasculitis with rituximab-associated hypogammaglobulinemia: infection outcomes and disease course in a retrospective cohort

**DOI:** 10.3389/fneph.2026.1861999

**Published:** 2026-08-04

**Authors:** Matthew Gross, Aditi Singh, Raj Roshan, Antoine Azar, Duvuru Geetha

**Affiliations:** Johns Hopkins Medicine, Johns Hopkins University, Baltimore, MD, United States

**Keywords:** ANCA, immunoglobulin deficiency, immunoglobulin replacement therapy, immunomodulation, rituximab, vasculitis

## Abstract

**Objective:**

To evaluate whether immunoglobulin replacement therapy (IgR), initiated for infection prophylaxis in patients with ANCA-associated vasculitis (AAV) receiving rituximab, is associated with changes in infection burden, disease activity, and the ability to de-escalate immunosuppression.

**Methods:**

We performed a retrospective single-center study of 16 patients with AAV treated with rituximab who developed secondary hypogammaglobulinemia (IgG <600 mg/dL) and subsequently received IgR between 2014 and 2024. Patients receiving IgR for primary immunodeficiency were excluded. Data collected included demographics, ANCA serotype, relapse history, infection burden, immunosuppressive regimen, and serum IgG levels before and after IgR. Primary outcomes were infections and disease relapse before versus after IgR initiation. Secondary outcomes included changes in serum IgG and rituximab use.

**Results:**

Prior to IgR, mean IgG was 287 ± 98 mg/dL, 14/14 patients tested had impaired vaccine responses, and 13/16 (81%) experienced recurrent or severe infections. Seven patients (44%) had at least one disease relapse while on rituximab maintenance. After IgR, mean IgG increased to 1018 ± 234 mg/dL, with normalization in 14/15 (93%). Recurrent or severe infections decreased to 1/16 (6%). Following IgR initiation, no disease relapses were observed during a median follow-up of 29.0 months (IQR 14.5-61.4; range 2.8-127.8), despite rituximab discontinuation in 10 patients and dose-interval extension in the remaining 6 patients. IgR was discontinued in 3 patients without relapse.

**Conclusion:**

In this cohort of AAV patients with rituximab-associated hypogammaglobulinemia, IgR demonstrated improved immunoglobulin levels and reduced infection burden and was associated with sustained disease remission despite immunosuppression de-escalation. Further studies are needed to determine whether IgR influences disease activity in AAV.

## Introduction

Antineutrophil cytoplasmic antibody (ANCA)-associated vasculitis (AAV) is a systemic small-vessel vasculitis characterized by a relapsing-remitting course that frequently necessitates prolonged immunosuppression. Rituximab (RTX) has become a cornerstone in both induction and maintenance of remission in AAV, offering a targeted approach through B-cell depletion (1). However, prolonged RTX use is associated with secondary hypogammaglobulinemia, impaired vaccine responses, and increased susceptibility to infections (2, 3). This remains an underrecognized adverse event in the treatment of AAV, with over 40% of patients experiencing moderate to severe hypogammaglobulinemia (4). This is further compounded using high-dose glucocorticoids (5). Intravenous immunoglobulin (IVIG) has been associated with decreased disease activity and ANCA titers and may be considered for patients with persistent disease activity, particularly those considered at increased risk for infection (6, 7). In one study, a single dose of IVIG (2 g/kg) reduced disease activity for up to 3 months in patients with persistent AAV (8). In another study, repeated IVIG administered as adjunctive therapy maintained clinical improvement for up to 2 years in patients with refractory or relapsing disease (9). Immunoglobulin replacement therapy (IgR), including IVIG and subcutaneous (SCIG) routes, is recommended for use for infection prophylaxis in hypogammaglobulinemic states (10). Whether IgR can also modulate immune disease activity and allow for de-escalation of immunosuppression in AAV remains unknown.

## Patients and methods

We conducted a retrospective study of patients with AAV who were treated with RTX and subsequently received IgR at our center between 2014 and 2024. Inclusion criteria were: (1) diagnosis of AAV confirmed by clinical criteria and/or biopsy; (2) treatment with RTX for induction or maintenance; (3) documented hypogammaglobulinemia (serum IgG <600 mg/dL); and (4) at least one dose of IgR. We excluded patients receiving IgR for primary immunodeficiency. Data collected included demographics, ANCA serotype, disease relapse history, infection burden, immunosuppressive regimen, and serum IgG levels pre- and post-IgR. Outcomes included relapse rate and infection burden before and after IgR initiation, change in serum IgG, and adjustments to RTX dosing. Infection episodes included bacterial, viral, and fungal infections, such as pneumonia, upper respiratory tract infections, sinusitis, influenza, COVID-19, herpes zoster, and urinary tract infections. Most infections were microbiologically confirmed; however, some common outpatient infections, particularly viral upper respiratory tract infections and sinusitis, were diagnosed clinically by the treating physician without microbiologic confirmation. Both inpatient and outpatient infections were included. Severe infections were defined as infections requiring hospitalization and/or intravenous antimicrobial therapy. Recurrent or severe infection burden was determined based on clinician documentation prior to and following initiation of immunoglobulin replacement therapy.

Disease relapse was defined as recurrent clinical manifestations of active ANCA-associated vasculitis requiring escalation of immunosuppressive therapy, including initiation or intensification of glucocorticoids, rituximab, or other immunosuppressive agents. Relapses were determined through retrospective chart review. Formal Birmingham Vasculitis Activity Score (BVAS) assessments were not routinely performed in clinical practice and therefore were not available for analysis.

Median age at diagnosis was 53 years (range 17-80), 81% were female, and all were Caucasian. Eleven patients were PR3-ANCA positive, three were MPO-ANCA positive, and two were negative for both PR3 and MPO antibodies. Seven patients (44%) had a history of relapsing disease, and 13 (81%) had recurrent or severe infections prior to IgR initiation. The median duration from AAV diagnosis to IgR initiation was 76.4 months (IQR 51.7–102.2), while the median follow-up after IgR initiation was 29.0 months (IQR 14.5–61.4).

## Results

### Immunoglobulin and infection outcomes

Mean (SD) serum IgG prior to IgR was 287 (98) mg/dL. Of the 15 patients tested for antibody response 4 weeks after administration of Pneumococcal polysaccharide vaccine-PPSV-23, 9 had no antibody response and the remaining 6 had diminished antibody response. Following IgR initiation (SCIG in 15 cases, IVIG in one), mean serum IgG rose to 1018 (234) mg/dL, and all 15 tested patients achieved significant improvement in IgG levels. Infections fell dramatically: only one patient (6%) experienced a severe infection post-IgR, compared with 13 patients (81%) pre-IgR (**Table 1**).

**Table 1:** Clinical outcomes before and after IgR.

Outcome	Before IgR	After IgR
Serum IgG, mean (SD), mg/dL	287 (98)	1018 (234)
Recurrent/severe infections	13/16 (81%)	1/16 (6%)
Relapses	7/16 (44%)	0/16 (0%)
RTX discontinued or interval extended	—	16/16 (100%)
Remission at last follow-up	—	16/16 (100%)

### Disease activity and immunosuppression after IgR

Before initiation of IgR, 7 of 16 patients (44%) experienced at least one disease relapse while receiving rituximab-based maintenance therapy. Following IgR initiation, no relapses were observed over a mean follow-up period of 1222 days (**Table 1**).

Adjustments to immunosuppression were common after IgR initiation. Rituximab was discontinued entirely in 10 of 16 patients (63%), while the dosing interval was extended in the remaining 6 patients (38%). At last follow-up, all 16 patients (100%) remained in clinical remission despite these reductions in immunosuppression. IgR therapy itself was eventually discontinued in 3 patients (19%) without subsequent relapse.

## Discussion

These findings suggest a dual association of IgR in patients with AAV and RTX-associated hypogammaglobulinemia. Not only did IgR reduce infection burden, but it was also associated with sustained disease remission despite tapering or cessation of RTX. IVIG has numerous immunomodulatory effects including suppression of proinflammatory cytokines, modulation of Fc receptor expression, and altering complement activation (11–13). It is an established treatment for other autoimmune diseases such as myasthenia gravis, immune thrombocytopenia, and chronic inflammatory demyelinating polyneuropathy. However, the potential for IgR to maintain remission in AAV has not been thoroughly studied. Prior studies have explored high-dose IVIG as an immunomodulatory adjunct in AAV, particularly in refractory or relapsing cases, with reported efficacy in inducing remission; however, these studies administered IVIG at doses higher than those typically used for replacement therapy (8, 9, 14). While direct causality cannot be inferred from our findings, the observation of sustained disease remission with replacement dose IgR despite immunosuppression de-escalation warrants further investigation.

This study is limited by its retrospective nature, single-center scope, lack of a control group, and small sample size composed of entirely Caucasian and predominantly female patients. In addition, IgR was initiated based on clinical concern for secondary antibody deficiency, impaired vaccine responses, and recurrent or severe infections, creating potential referral and treatment-selection bias. Patients who received IgR may therefore have differed systematically from RTX-treated patients with AAV who did not receive IgR, including differences in infection susceptibility, immunosuppressive exposure, disease severity, comorbidities, clinician referral patterns, and likelihood of immunology evaluation In addition, patients contributed substantially longer observation time prior to IgR initiation than after IgR initiation, and therefore comparisons of infection and relapse frequencies should be interpreted descriptively rather than as formal incidence rate comparisons. Because this was a retrospective study with heterogeneous rituximab treatment strategies, we cannot distinguish the independent contribution of immunoglobulin replacement from the durable immunologic effects of prior rituximab therapy. Accordingly, our findings should be interpreted as demonstrating an association between IgR and sustained remission during immunosuppression de-escalation rather than evidence of a direct disease-modifying effect of IgR. Nevertheless, the consistent observation of durable remission despite significant reduction or cessation of RTX is notable. The normalization of IgG and resolution of recurrent infections support the effectiveness of IgR in secondary antibody deficiency. Further prospective studies are needed to evaluate the relationship between IgR and disease activity in autoimmune vasculitis, including its effects on B-cell repopulation, cytokine profiles, and long-term remission.

## Data Availability

The raw data supporting the conclusions of this article will be made available by the authors, without undue reservation.
